# Bringing Fundamental Insights of Induced Resistance to Agricultural Management of Herbivore Pests

**DOI:** 10.1007/s10886-023-01432-3

**Published:** 2023-05-04

**Authors:** Erik H. Poelman, Mitchel E. Bourne, Luuk Croijmans, Maximilien A. C. Cuny, Zoë Delamore, Gabriel Joachim, Sarah N. Kalisvaart, Bram B. J. Kamps, Maxence Longuemare, Hanneke A. C. Suijkerbuijk, Nina Xiaoning Zhang

**Affiliations:** Laboratory of Entomology, Wageningen University, P.O. Box 16, 6700AA Wageningen, the Netherlands

**Keywords:** Induced direct and indirect resistance, Plant defence, Sustainable ecology-based cropping systems, Pest management strategies

## Abstract

In response to herbivory, most plant species adjust their chemical and morphological phenotype to acquire induced resistance to the attacking herbivore. Induced resistance may be an optimal defence strategy that allows plants to reduce metabolic costs of resistance in the absence of herbivores, allocate resistance to the most valuable plant tissues and tailor its response to the pattern of attack by multiple herbivore species. Moreover, plasticity in resistance decreases the potential that herbivores adapt to specific plant resistance traits and need to deal with a moving target of variable plant quality. Induced resistance additionally allows plants to provide information to other community members to attract natural enemies of its herbivore attacker or inform related neighbouring plants of pending herbivore attack. Despite the clear evolutionary benefits of induced resistance in plants, crop protection strategies to herbivore pests have not exploited the full potential of induced resistance for agriculture. Here, we present evidence that induced resistance offers strong potential to enhance resistance and resilience of crops to (multi-) herbivore attack. Specifically, induced resistance promotes plant plasticity to cope with multiple herbivore species by plasticity in growth and resistance, maximizes biological control by attracting natural enemies and, enhances associational resistance of the plant stand in favour of yield. Induced resistance may be further harnessed by soil quality, microbial communities and associational resistance offered by crop mixtures. In the transition to more sustainable ecology-based cropping systems that have strongly reduced pesticide and fertilizer input, induced resistance may prove to be an invaluable trait in breeding for crop resilience.

In addition to a constitutive first layer of chemical and morphological defences, virtually all plant species perceive and respond to risks of herbivore attack (Acevedo et al. 2015; Bonaventure et al. 2011). Plants perceive forthcoming attack by, for example, volatile information of neighbouring plants that are under herbivore attack (Moreira and Abdala-Roberts 2019), or cues derived from egg deposition by herbivores on or in plant tissues (Griese et al. 2021). These cues and those associated with actual plant damage by herbivory, trigger signal transduction cascades regulated through phytohormones that lead to chemical and morphological changes in the plant phenotype (Howe and Jander 2008; Pieterse et al. 2012). These so-called herbivore-induced plant responses may result in induced resistance by reducing herbivore food plant acceptance or herbivore performance and/or by enhancing top down control of herbivores by predators. The induced level of resistance is termed an induced defence when it prevents the individual plant from a fitness loss, for example expressed in its number and quality of seeds produced (Erb 2018; Karban and Baldwin 1997; Poelman 2015). Natural selection favours induced plant defences when the plant: i) correctly evaluates the risk of herbivore attack, ii) accurately adapts its induced response for the type of herbivore attack, and iii) reduces herbivory fast enough to counter its associated fitness costs (Agrawal 2011; Karban and Orrock 2018). In many plant life-histories, inducibility of resistance optimizes how plants balance growth and defence to maximize fitness (Agrawal and Züst 2017; Herms and Mattson 1992). Recent advances in studies on herbivore induced plant responses highlight that the fitness benefit (and cost) of induced responses may exceed far beyond an interaction with a specific herbivore (Mertens et al. 2021a, b; Strauss et al. 2002). Any changes to plant phenotype by herbivore induced responses may alter the plant interaction with other community members and thereby affect the plant fitness outcome of the induced response (Kessler and Baldwin 2004; McArt et al. 2013). The induced response may even include a spatial component allowing plants to communicate with and enhance performance of other plant individuals (Braasch and Kaplan 2012).

The spatial component of how induced resistance affects the performance of multiple plants in a stand, may in particular provide opportunities to agriculture where yield of the full crop is more important than performance of individual plants. In addition, inducible resistance may enhance crop performance by balancing growth and defence allocation trade-offs to stimulate growth in the absence of herbivory (Agrawal and Züst 2017). Moreover, enhancing herbivore induced plant volatile emission in crop cultivars may promote biological pest control through recruitment of natural enemies to the crop (Croijmans et al. 2022; Poelman et al. 2009). Inducible responses may also allow crops to effectively deal with multiple co-occurring biotic and abiotic stresses by enhancing plasticity to stress across the growing season. Especially in the transition to more sustainable cropping systems that reduce pesticide and fertilizer input, induced resistance may be key to enhance crop resilience. This is because cropping systems without chemical control of pests, typically harbour multiple herbivore pests that need to be controlled by ecological processes in which induced plant responses may be capitalized (Bourke et al. 2021; Divekar et al. 2022). However, current crop cultivars have not been bred for induced resistance and resilience to multi-stress situations.

Here we explore how knowledge on natural selection of induced resistance translates to agricultural perspective where natural selection is replaced by artificial selection and fitness of an individual replaced by crop yield (Fig. 1). We review the fundamental theories of induced resistance and how a community perspective is providing new insights into natural selection on inducible traits. We then argue how these theories can be applied to agriculture when exploiting inducible traits. The emerging future perspective is that induced resistance offers strong potential to enhance resistance and resilience of cropping systems. Induced resistance should thus be seen as an integral part of agriculture in transition to more sustainable ecology-based cropping systems.

**Fig. 1:**
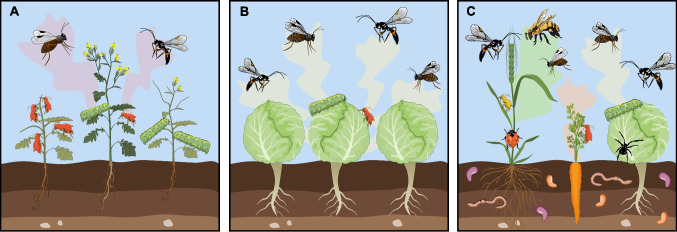
Plant inducible resistance inspired from wild systems and translated into agriculture. (**A**) Induced resistance in wild system is driven by natural selection on individual plants to subsist and compete in a multi- stressor environment. (**B**) Incorporating inducible traits in agriculture may enhance plasticity of the plant stand to cope with multiple (a)biotic challenges while sustaining yield. (**C**) Including induced resistance in crop mixtures may harness the resilience of the cropping system by enhancing soil quality, increasing the diversity of natural enemies, pollinators and diluting pest pressure

## Fundamental Theory of Induced Resistance

Evolution of induced resistance is explained by three non-mutually exclusive hypotheses that build from the focus on individual plants to a plant community perspective (Karban 2020; Kessler 2015).

The first hypothesis, optimal defence theory, argues that individual plants manage the metabolic expenses to mount resistance to herbivores as a cost saving strategy (Zangerl 2003; Agrawal and Züst 2017). The metabolic costs of resistance are traded-off against investment in growth and reproduction. With certainty and high risk of herbivore attack, plants should build a strong layer of constitutive resistance to reduce consumption of their tissues (Agrawal et al. 2006; Agrawal and Züst 2017). To reduce these metabolic expenses, plants allocate their resistance to the most valuable tissues such as the main growth meristem, tap root, flowers and seeds (Godschalx et al. 2016). Moreover, the allocation of resources to resistance typically varies across plant phenology depending on the risk of herbivore attack, the relative costs of losing tissues to herbivory, and the photosynthetic capacity or metabolic reserves to invest in resistance traits (Barton and Boege 2017; Zangerl and Rutledge 1996). For example, seedlings may be heavily defended because any amount of herbivory would strongly reduce life expectancy of the plant, whereas the same amount of herbivory can be proportionally tolerated without significant loss of plant fitness when the plant reaches substantial biomass (Barton and Boege 2017; Bustos-Segura et al. 2022). When herbivore attack is uncertain, induced resistance allows plants to only pay the metabolic investment for resistance when it is actually under attack by herbivores. It may favour metabolic investment in growth and reproduction when herbivores are absent. Induced resistance may even further optimize metabolic expenses to resistance when these accurately identify and respond to herbivore location and identity (Malook et al. 2022; Stam et al. 2014). Cell wall disruption by herbivory causes damage-associated molecular patterns (DAMPS) that allow plants to detect where and which plant tissues are under attack. Moreover, the identity of a specific herbivore can be recognized by herbivore-associated molecular patterns (HAMPS) for example by those contained in herbivore saliva (Acevedo et al. 2015; Duran-Flores and Heil 2016; Erb and Reymond 2019; Schuman and Baldwin 2016).

The second hypothesis, moving target theory, takes the evolutionary perspective of herbivore adaptations to plant resistance. It argues that induced plant responses make plants moving targets to herbivore adaptations that counter plant resistance (Adler and Karban 1994). Temporal and spatial variations in resistance make food plant quality to herbivores more variable and cause difficulties for herbivores to match their physiology with the mix of primary and secondary metabolites of the plant (Petrén et al. 2023). Insect herbivores may be hampered in their detoxification capacity to plant chemical resistance, digestion of plant tissue to acquire nutrients, or food plant acceptance for oviposition or feeding when plant nutritional quality is highly variable (Kessler and Kalske 2018; Stork 2009). Directional selection for adaptation to a specific resistance trait is distorted by large variation in the same or different traits that exert selection on the herbivore. Therefore, the rate at which herbivores adapt to plant resistance can be reduced by spatial and temporal differences in resistance through variations in genotype and induced plasticity within a plant population (Kessler and Kalske 2018).

The third hypothesis, information transfer theory, argues that induced responses to herbivory create reliable information to/from community members that i) provide the emitting plants themselves with enhanced resistance or ii) reduce herbivory on related plant individuals in the neighbourhood (an inclusive fitness benefit) (Kessler 2015; Morrell and Kessler 2017). Herbivore induced plant responses generally include release of chemical compounds such as volatiles or root exudates into the environment. These chemical compounds may provide other community members such as herbivores, natural enemies or neighbouring plants with information about herbivore attack. The release of these chemical compounds may first of all directly benefit the emitting plant when it i) alters food plant acceptance by subsequently colonizing herbivores, ii) causes current herbivore attackers to spread their damage more evenly over the plant reducing pressure on valuable tissues, or iii) causes herbivores to move to neighbouring plants (Karban and Yang 2020; Rubin et al. 2015; Veyrat et al. 2016). Moreover, the information may be perceived by natural enemies of the herbivores as reliable and detectable cue of prey presence. Recruitment of natural enemies by herbivore induced plant volatile information may enhance top-down control of herbivores to reduce plant damage and serve as an indirect defence strategy (Pearse et al. 2020; Schuman et al. 2012). The plant may use herbivore induced volatile information to inform its own tissues or neighbouring plants of the risk of herbivore attack. Plant tissues that are spatially close but distantly connected by the sap stream, like branches of a tree, may be more rapidly informed by herbivore induced volatiles (Frost et al. 2007). The same volatile cues may also serve as a source of information for related neighbouring plants to inform them of the risk of herbivore attack. Neighbouring plants that perceive the volatile information may prepare for subsequent attack by entering a primed state that allows a more rapid and vigorous response when the herbivore arrives (Conrath et al. 2006; Douma et al. 2017; Kalske et al. 2019; Mauch-Mani et al. 2017). Because related plant individuals (kin) are often strong responders to the information, herbivore induced information transfer may provide emitters with the potential to especially protect kin (Kalske et al. 2019). Information transfer to enhance inclusive fitness by protecting kin may be most pronounced in clonal networks. In these networks, direct neighbours are often kin. When one individual responds to herbivory, herbivores often move to less defended neighbours. However, these neighbours may already be well prepared for herbivore attack and their vigorous responses cause herbivores to move further away outside the circle of influence by the emitter plant (Morrell and Kessler 2017). The inclusive fitness benefit may therefore even be enhanced by spatial processes of information transfer and herbivore movement and thus stimulate selection for induced resistance (Anten and Chen 2021).

### Costs and Benefits of Induced Resistance in Community Perspective

Each of the hypotheses on the evolutionary drive behind induced resistance should be placed in a community perspective to fully understand how benefits of induced resistance outweigh costs. The primary costs associated with induced resistance are not the metabolic costs, but so-called ecological costs that arise through the effect of the induced phenotype on ecological interactions with community members (Heil 2002; Strauss et al. 2002).

Inducibility of resistance traits does allow plants to save metabolic costs of resistance in the absence of herbivores, but also creates costs due to a lag time between recognition of herbivore attack and the mobilisation of effective resistance (Karban 2020; Mertens et al. 2021a). During the lag time, the plant is less well defended against herbivore attack. Moreover, the benefit of mounting a specific response to the current attacker has strong implications for the physiological capabilities of plants to respond to subsequently arriving herbivores. Mobilization of resistance through phytohormonal signalling and broad transcriptional reprogramming to deal with the current attacker may impair plants in maximizing resistance or even result in susceptibility to other herbivores (Dong et al. 2020; Fernandez de Bobadilla et al. 2022; Stam et al. 2014). First, the cross talk between different signal transduction routes to deal with different herbivore attackers allows plants to fine tune responses to multiple attackers. However, at the same time, regulation of resistance to one attacker may impair accurate induced resistance to subsequent attacks due to antagonistic interactions among signal transduction routes (Pieterse et al. 2012; Vos et al. 2013). Second, inducibility makes plants prone to herbivores hijacking plant plasticity for their own benefit. For example, aphids have been hypothesized to manipulatively induce the Salicylic Acid (SA) pathway of plants to use antagonistic cross talk with the Jasmonic Acid (JA) pathway to suppress plant resistance to the aphids (Selig et al. 2016). Along the same lines, microorganisms associated with herbivores may supress or reduce plant responses to herbivory (Minard et al. 2022; Zhu et al. 2018a). Moreover, parasitic organisms that live inside herbivores, such as the larvae of parasitoids, alter herbivore-associated molecular pattern composition such as elicitors in saliva potentially affecting plant recognition of the identity of the herbivore attacker (Tan et al. 2018, 2019; Zhu et al. 2018b).

Similar to constitutive expression of resistance traits, induction of specific traits may lead to resistance against one herbivore but to susceptibility for others (Ali and Agrawal 2012). Plant lineage specific resistance traits such as glucosinolates in Brassicaceae and nicotine in *Nicotiana* are defensive against generalist herbivores. However, specialist herbivores use these compounds in food plant recognition and acceptance (Ali and Agrawal 2012). Moreover, the specific response to one herbivore causes herbivore specific priority effects in herbivore community assembly (Fukami 2015; Mertens et al. 2022). Historical contingency or priority effects of plant responses to current attack mediate interactions with other herbivores in the community and influence the pattern of future attack (Mertens et al. 2021a, b; Poelman et al. 2008). These so-called plant-mediated species interactions link above and belowground communities and may result in life-time consequences for community composition on individual plants (Kessler and Halitschke 2007; Stam et al. 2014). Moreover, plant-mediated interactions extend to beneficial interactions such as flower visitation by pollinators. Root, leaf and flower feeding by herbivores also induces changes in flower traits such as colour, scent and nectar rewards that affect recruitment of pollinators with direct consequences to reproductive success (Rusman et al. 2019). Plant mediated species interactions have been identified to affect plant fitness and may thereby affect evolution of induced resistance (McArt et al. 2013; Poelman and Kessler 2016).

The costs and benefits of the induced resistance are further highly dependent on the plant community context. The costs of investment in resistance against herbivores depends on the severity of plant-plant competition for light and nutrients (Agrawal and Züst 2017; Herms and Matson 1992). In high plant density, or competitive environments, loss of photosynthetic capacity by herbivory as well as metabolic costs of resistance that reduces growth potential enhance the likelihood of being outcompeted by neighbouring plants. These costs and benefits can, for example, be modulated by variations in herbivore traits like the timing and feeding location of herbivores in the plant (de Vries et al. 2017, 2019). The use of volatile information as part of the induced response to herbivory affects how kin and non-kin neighbours experience ecological costs and benefits of induced resistance of a focal plant (Bilas et al. 2021). The spatial component of induced responses may thus extend induced resistance with an inclusive fitness component of how fitness of other individuals is affected by induced resistance. In their induced resistance to one herbivore, plants should therefore balance the physiological and ecological consequences of the response in community context. This may include that plants anticipate patterns of attack by different species in their response to current herbivore attack (Mertens et al. 2021b; Fernandez de Bobadilla et al. 2022). The evaluation whether an induced response indeed leads to a fitness benefit should be made in the context of the impact of the response on the full community associated with the individual plant (Erb 2018; Poelman 2015).

### Incorporating Inducible Traits in Agriculture

Industrialized cropping systems maximize yield of monocultures by high input of fertilizers and pesticides. In these single species stands, crop genetic uniformity results in high efficiency of mechanized crop management. Although industrialized farming systems have succeeded in supplying the global food demand, they generate negative outcomes that are incompatible with planetary sustainability goals. These negative outcomes include greenhouse gas emissions, vulnerability of agroecosystems to environmental conditions such as drought and rainfall, productivity loss through degradation of soils, simplification of the agroecological landscape, and contamination of soil and water with pesticides and fertilizers (Garibaldi et al. 2011a, b). The latter two in turn cause biodiversity loss, which includes loss of ecosystem services such as pollination (Stanley et al. 2015) and control of pests and pathogens. Moreover, these agricultural systems are prone to build up of pesticide resistance in insect herbivores and rapid adaptation of pests and pathogens to crop resistance traits. Often, breeding practices are geared towards maximizing yield, uniformity in growth and resistance traits of a plant stand. Crop domestication has reduced chemical resistance against herbivorous insects (Chen et al. 2015) and these effects are most consistent for reproductive plant organs as well as the organs harvested for human consumption (Whitehead et al. 2017). Along with reduced chemical resistance, domesticated crops may have reduced induced direct resistance against insect herbivores (Moreira et al. 2018). Moreover, domestication may have caused loss in strength of induced indirect resistance by volatile attraction of natural enemies of herbivores (Benrey 2023; Degenhardt et al. 2009). Although crops still poses a level of induced resistance, genetically and phenotypically uniform cropping systems largely ignore the potential of trait variation that can be achieved by induced resistance (Table 1).

**Table 1:** Hypotheses of induced resistance translate to opportunities in agriculture

	Hypotheses associated with wild systems	Opportunities for cultivated systems
Optimal defence	• Cost saving strategy to balance growth, defence and reproduction • Accurately respond to multiple simultaneous or sequential stresses • Protection of most valuable tissue depending on life stage	• Decouple growth and defence to maintain crop uniformity and increase plasticity in resistance • Strengthen cross-resistance and reduce potential for induced susceptibility to multiple attackers • Select for altered allocation of defensive compounds to protect harvestable product
Moving target	• Genotypic variation and plasticity in plant resistance reduces speed of herbivore evolutionary adaptations	• Select for inducible traits to maximize plasticity in resistance • Use of crop mixtures for genotypic and phenotypic variation in agricultural field
Information transfer	• Volatile communication between tissues for quick transmission of information • Plant-plant communication • Deter subsequent herbivores and attract natural enemies	• Utilize plant-plant and tissue communication for associational resistance between crops • Select for cues that maximize the potential for biological control

Strong direct evidence of benefits from plant induced responses to herbivory are found for crops of which specific organs are harvested as product such as tubers or seeds. Aboveground attack of potato plants by aphids may enhance tuber production, because plants reprogramme their investment in tubers upon induced responses to aphid attack (Poveda et al. 2010). Similarly, when insect herbivores damage main growth meristems, activation of growth in other meristems may result in larger number of side branches that increases flower and seed production (Garcia and Eubanks 2019). The reallocation of resources to tubers and overcompensation of flower production directly follow from predictions of optimal defence theory in which plasticity in resistance allows for reprogramming of growth and defence under herbivore attack (Gagic et al. 2016). Allowing for plasticity in resistance to herbivores will also benefit the potential of crops to deal with abiotic conditions. Induced responses to abiotic and biotic stress are regulated by similar signal transduction routes that also cross communicate to fine tune responses to simultaneous abiotic and biotic stress (Nguyen et al. 2016). Induced resistance to herbivore attack may reduce the vulnerability of crops to drought and heavy rainfall, because crops may become more resilient to multi-stress conditions when they are plastic in resistance.

In production systems with reduced to no pesticide use, herbivore communities will be diverse and crops often deal with sequential and simultaneous attack by different herbivore species. Herbivore induced resistance may be critical in dealing with uncertainty in the type, timing, order and severity of attack by herbivores that demands plasticity in resistance. Enhancing crop performance through induced resistance can be reached by breeding for plasticity per se, to allow plants to respond to specific herbivore attackers or patterns of herbivore attack. This may incorporate anticipatory responses of plants in which an induced response to the current attacker includes preparation for likely future attack by other herbivore species (Mertens et al. 2021a). In these strategies, a selective approach may be required to reduce negative impacts of induced susceptibility to subsequent herbivore attack. A promising practical example of induced resistance may be found in vaccination strategies, where less damaging herbivore species are reducing the impact of the most ravaging pest herbivores (Kessler and Baldwin 2004). In wild tobacco, plant induced responses to myrid bug feeding reduced the colonization of plants by the Tobacco hornworm that is a voracious feeder. Although it may seem counterintuitive to farmers, some level of herbivory by myrids may actually enhance crop performance by protecting the crop from more severe damage (Kessler and Baldwin 2004). Plant vaccination can also be achieved directly by farmers, either by spraying inducible compounds such as jasmonic acid on the leaves (War et al. 2011; Zhang et al. 2023), or by releasing omnivorous herbivores that feed on the leaves (with a low impact on yield) until they find their herbivorous prey (Omer et al. 2000; Pappas et al. 2015). However, induced responses to some pests may also result in enhanced susceptibility to herbivore attack and reduced biological pest control. Vaccination strategies thus require detailed understanding of interactions among pests.

There may be potential of herbivore induced resistance to stimulate crop yield through interactions with pollinators, although the outcome of induced resistance on pollination and crop yield is highly variable (Kessler and Chauta 2020). In some crops such as strawberry, induced responses to herbivory have been found to negatively affect pollination (Muola et al. 2017). In contrast, in oilseed rape these interactions increased yield by overcompensation to herbivory and increases in pollinator attraction (Gagic et al. 2016). Root, leaf, flower and fruit feeding herbivores may affect pollinator communities visiting plants, as well as the duration of visits, time spent per flower and thereby effectiveness of pollination. Each pollinator species may respond differently to herbivore induced changes in flower traits (Kessler and Baldwin 2007; Rusman et al. 2019). Induced increases in concentrations of secondary chemistry in flowers may be a driver for specialization in the plant-pollinator relationship (Stevenson et al. 2017), or variation in effects on pollinator species do result in shifts in pollinator community composition without reducing overall pollinator visits (Chrétien et al. 2021). Traces of nicotine and caffeine in floral nectar rewards increase visitation by bees (Singaravelan et al. 2005). Caffeine has been found to strengthen honeybee memory of floral reward (Wright et al. 2013). This suggests that herbivore induced changes in concentrations of these compounds may increase pollination (Thomson et al. 2015). Foremost, replacing crop protection by pesticides with ecological interactions driven by induced resistance will indirectly promote pollination services (Stanley et al. 2015). With reduced pesticide use, crops will benefit from pollination by a diverse community of wild pollinator species. Their pollinator services often exceed the services by managed species such as honeybees (Adler and Hazzard 2009; Fijen et al. 2018; Garibaldi et al. 2011a, b). In particular, a diverse pollinator community will increase resilience in agricultural systems as the work of many pollinator species is complementary (Blüthgen and Klein 2011; Sapir et al. 2017). By adopting more plant diversity on and surrounding agricultural fields (Balfour and Ratnieks 2022; Bänsch et al. 2021; Koski et al. 2021; Kovács-Hostyánszki et al. 2017; Nicholls and Altieri 2013) and banning pesticide use, industrial agriculture can be transformed to have a more mutualistic interaction with wild pollinators and continue to benefit from their services (worth €153 billion (Gallai et al. 2009).

Biological control by attracting natural enemies inherently relies on inducibility for information mediated interactions between plants and natural enemies. To effectively locate hosts or prey, natural enemies use reliable information on host presence. Reliability is not achieved by constitutive high expression of for example volatile information that attracts natural enemies. False information on the presence of a reward may discourage natural enemies to respond to cues that should attract natural enemies (Beale et al. 2006; Kunert et al. 2010; Bruce et al. 2015). The association between information and reward is essential to help natural enemies optimize foraging efficiency and maximize biological pest control. Herbivore induced plant volatiles (HIPVs) enable crops to release information of herbivore presence only upon actual herbivore attack. However, selective breeding has indirectly resulted in reduced attraction or detectability of HIPVs by natural enemies (Li et al. 2018). There is, therefore, strong potential to strengthen this cue by exploiting variation in inducibility of volatile emission by crop accessions (Pappas et al. 2017). For example, Maize varieties that emit (*E*)-*β*-caryophyllene in response to root feeding by the western corn rootworm attract entomopathogenic nematodes that infect and kill the root pest. These interactions could be restored through transformation breeding of this trait into North-American varieties that had lost the ability to produce (*E*)-*β*-caryophyllene (Degenhardt et al. 2009). In cabbage some cultivars more strongly respond to feeding by *Pieris* caterpillars by release of volatiles that are more attractive to the parasitoids than HIPVs from other cabbage cultivars (Croijmans et al. 2022; Poelman et al. 2009). In the field, this cabbage cultivar not only attracts parasitoids over a longer distance (Aartsma et al. 2019), the attraction also leads to enhanced parasitism rates of *Pieris* caterpillars on this cultivar (Poelman et al. 2009). The success of such strategies to attract natural enemies towards pest infested agricultural crops will also depend on available resources in the field and landscape. Landscapes that provide natural enemies with carbohydrates and shelter will likely contain greater source populations. Inclusion of crops with rewarding inducible traits, like extra-floral nectaries (Mathur et al. 2013), could further enhance survival and retention of natural enemies within the field (Kaiser et al. 2017; Stenberg et al. 2015). An important balancing factor that may reduce efficiency of biological control by parasitoids is the role of plant resistance in host immune responses to parasitism. High levels of chemical resistance may reduce the immune capacity of herbivores against parasitism and enhance survival of herbivores after parasitoid attack (Benrey 2023). These findings highlight the potential to breed for inducibility of volatile emission in crops to strengthen biological control, especially when these are holistically combined with other cropping system diversification measures.

### Including Induced Resistance in Crop Mixtures

Nevertheless, the increase in herbivore diversity attacking crops after relief of pesticide use may not be fully compensated by inducible resistance. Multi-stress situations, biotic and abiotic, may require plasticity in response to stress combinations and sequences, but also to balance interactions with beneficial organisms. To tailor induced resistance in a monocultural crop to a full community perspective may be challenging. Even more so, abiotic conditions such as water and nutrient availability may directly impact the vigour of inducibility of the crop, reducing crop potential to respond to herbivory. Crop mixtures and crop rotation strategies may be required to harness the resilience of a cropping system and promoting benefits of induced resistance (Koski et al. 2021).

First, crop rotation, reduced tillage and intercropping may enhance soils quality. These measures stimulate macrofauna in the detritivore web that may promote nutrient availability, reduce abiotic perturbations and thereby enhance the potential of crops to express their full potential of induced resistance (Beillouin et al. 2019). Moreover, soil microbiomes and their associations with roots have been identified to enhance resistance through induced plant responses (Cameron et al. 2013; Lee-Diaz et al. 2021). This includes induced indirect resistance to attract natural enemies (Malone et al. 2022). Enhancing beneficial microbes in the soil may therefore enhance benefits of induced resistance.

Second, crop mixtures may enhance the potential of information transfer as means to reduce pest pressure. Push–pull systems developed for sustainable pest management in maize, identify how associational resistance and induced indirect resistance by attraction of natural enemies may be combined to reduce crop damage by herbivores. By volatile emission, companion crops mask the presence of cash crops to insect herbivores or even repel insect herbivores out of the crop field. Inducibility of attractive volatiles to natural enemies in the cash crop may at the same time enhance biocontrol of the pest individuals that still colonize the cash crop (Chidawanyika et al. 2023; Khan et al. 2011, 2016). In addition, such cropping systems benefit from peripheral trap crops that attract the herbivore pest that can thereby be removed from the cropping system (Adler and Hazzard 2009).

Third, crop diversity may enhance food web stability by harbouring a more diverse community of natural enemies that control a diverse community of herbivores (Haddad et al. 2011a, b). Thus, cropping systems based on crop mixtures may provide the pool of natural enemies that can be attracted to specific pests on crops by induced resistance through volatile emission. There may, however, be a tipping point in the benefits of diversity for conservation biological control. Structural complexity of the cropping system may reduce volatile information transfer and impair movement by natural enemies, reducing the apparency of HIPVs (Bukovinszky et al. 2005; 2007). Crops with apparent and specific inducible cues might steer natural enemies towards a more diverse community of herbivores, despite the increased noise created by structural complexity of the plant community.

Fourth, the moving target theory predicts that induced resistance would reduce the rate and potential of insect herbivores to evolve adaptations to resistance traits. Even in monocultures with high genetic uniformity, induced resistance may reduce the potential of natural enemies to adapt to resistance traits. Enhancing crop genetic or species diversity will create an even more diverse patchwork of defence mechanisms that will further strengthen the resistance of cropping systems to counteradaptations by herbivores (Espinosa-Garcia 2022).

### Future Perspectives

Utilizing induced resistance is a promising strategy to aid the transition to sustainable ecology-based agriculture. Plasticity in resistance offers a way out in dealing with a large number of stressors (insect herbivores, abiotic stress), maximizing the impact of biological control, and making crops resilient to changes in the environment. Using induced resistance in agriculture clearly has implications for farming practices and requires in depth understanding of both the physiological regulation of inducible traits as well as its role in ecological interactions (Chrétien et al. 2021; Divekar et al. 2022).

Perhaps the most prominent challenge may be that inducibility could reduce uniformity in growth and ripening of the crop, enhancing variation in crop quality. This directly affects harvesting strategies as well as the marketing of the crop that needs to be sold with a larger distribution over quality classes. In depth understanding of how induced resistance is traded-off or coupled to growth and phenological trajectories of plants may provide breeders with opportunities to decouple growth-defence responses (Bourke et al. 2021; de Vries et al. 2017; Karasov et al. 2017).

Matching induced resistance traits with the local community of herbivores, challenges by abiotic conditions as well as landscape context that determines availability of natural enemies will require knowledge on local species interactions. Farmers themselves may need to increase the monitoring of crops when these are under attack by more numerous pest species. Such a monitoring challenge may to an extent be overcome by apps for species identification, although such apps could be improved by taking host plant into account in the search scope. At the same time, breeders may have to incorporate the context of community dynamics on a specific farm to advise for the use of induced resistance and/or combination of crop accessions or species that result in a resilient cropping system to local conditions. Soil quality and microbial community composition may prove to be critical in harnessing the vigour of inducible resistance (Cameron et al. 2013; Lee-Diaz et al. 2021). Crop breeding companies may become businesses that not only provide a product, but offer services known from biological control companies that offer knowledge and implementation strategy to secure crop resilience and resistance. This requires farmers and breeding companies to build new trust relationships that come with tailor made designs of pest management strategies.

To start breeding for induced resistance and understand the community consequences of inducibility in a crop, we may learn from wild plants. We require understanding of how inducibility matches with other life-history traits of plants. For example how mating system, constitutive defence, lifespan, or growth-defence trade-offs correlate with inducibility will identify the potential to select for inducibility in specific crops (Garcia et al. 2021; Johnson et al. 2015). At the same time we should unravel how inducible traits alter species interactions and what parts of community dynamics select for induced resistance in plants (Mertens et al. 2021a). These insights will allow us to match plastic resistance strategies with the complexity of ecological interactions that arise in sustainable agricultural practices. Combining both genetic diversity in cropping systems with inducible resistance will restore and strengthen ecosystem processes in agricultural fields and reduce pest prevalence (Bourke et al. 2021).

## References

[CR1] Aartsma Y, Leroy B, van der Werf W, Dicke M, Poelman EH, Bianchi FJJA (2019). Plant variation in herbivore-induced volatiles influences the spatial range of parasitoid attraction. Oikos.

[CR2] Acevedo FE, Rivera-Vega LJ, Chung SH, Ray S, Felton GW (2015). Cues from chewing insects – the intersection of DAMPs, HAMPs, MAMPs and effectors. Curr Opin Plant Biol.

[CR3] Adler FR, Karban R (1994). Defended fortresses or moving targets? Another model of inducible defenses inspired by military metaphors. Am Nat.

[CR4] Adler LS, Hazzard RV (2009). Comparison of perimeter trap crop varieties: Effects on herbivory, pollination, and yield in Butternut Squash. Environ Entomol.

[CR5] Agrawal AA, Lau JA, Hambäck PA (2006). Community heterogeneity and the evolution of interactions between plants and insect herbivores. Quarterly Rev Biol.

[CR6] Agrawal AA (2011). Current trends in the evolutionary ecology of plant defence. Funct Ecol.

[CR7] Agrawal AA, Züst T (2017). Trade-Offs Between Plant Growth and Defense Against Insect Herbivory: An emerging mechanistic synthesis. Annu Rev Plant Biol.

[CR8] Ali JG, Agrawal AA (2012). Specialist versus generalist insect herbivores and plant defense. Trends Plant Sci.

[CR9] Anten NPR, Chen BJW (2021). Detect thy family: Mechanisms, ecology and agricultural aspects of kin recognition in plants. Plant Cell Environ.

[CR10] Balfour NJ, Ratnieks FLW (2022). The disproportionate value of ‘weeds’ to pollinators and biodiversity. J Appl Ecol.

[CR11] Bänsch S, Tscharntke T, Gabriel D, Westphal C (2021). Crop pollination services: Complementary resource use by social vs solitary bees facing crops with contrasting flower supply. J Appl Ecol.

[CR12] Barton KE, Boege K (2017). Future directions in the ontogeny of plant defence: Understanding the evolutionary causes and consequences. Ecol Lett.

[CR13] Beale MH, Birkett MA, Bruce TJA, Chamberlain K, Field LM, Huttly AK, Martin JL, Parker R, Phillips AL, Pickett JA, Prosser IM, Shewry PR, Smart LE, Wadhams LJ, Woodcock CM, Zhang Y (2006). Aphid alarm pheromone produced by transgenic plants affects aphid and parasitoid behavior. Proc Natl Acad Sci USA.

[CR14] Beillouin D, Ben-Ari T, David Makowski D (2019). Evidence map of crop diversification strategies at the global scale. Environ Res Lett.

[CR15] Benrey B (2023). The effects of plants domestication on the foraging and performance of parasitoids. Curr Opin Insect Sci.

[CR16] Bilas RD, Bretman A, Bennett T (2021). Friends, neighbours and enemies: an overview of the communal and social biology of plants. Plant Cell Environ.

[CR17] Blüthgen N, Klein AM (2011). Functional complementarity and specialisation: The role of biodiversity in plant-pollinator interactions. Basic Appl Ecol.

[CR18] Bonaventure G, Van Doorn A, Baldwin IT (2011). Herbivore-associated elicitors: FAC signaling and metabolism. Trends Plant Sci.

[CR19] Bourke PM, Evers JB, Bijma P, van Apeldoorn DF, Smulders MJM, Kuyper TW, Mommer L, Bonnema G (2021). Breeding beyond monoculture: Putting the “Intercrop” into crops. Front Plant Sci.

[CR20] Braasch J, Kaplan I (2012). Over what distance are plant volatiles bioactive? Estimating the spatial dimensions of attraction in an arthropod assemblage. Entomol Exp Appl.

[CR21] Bruce TJA, Aradottir GI, Smart LE, Martin JL, Caulfield JC, Doherty A, Sparks CA, Woodcock CM, Birkett MA, Napier JA, Jones HD (2015). Pickett JA (2015) The first crop plant genetically engineered to release an insect pheromone for defence. Sci Rep.

[CR22] Bukovinszky T, Gols R, Posthumus MA, Vet LEM, Van Lenteren JC (2005). Variation in plant volatiles and attraction of the parasitoid Diadegma semiclausum (Hellén). J Chem Ecol.

[CR23] Bukovinszky T, Gols R, Hemerik L, Van Lenteren JC, Vet LEM (2007). Time allocation of a parasitoid foraging in heterogeneous vegetation: implications for host–parasitoid interactions. J Anim Ecol.

[CR24] Bustos-Segura C, González-Salas R, Benrey B (2022). Early damage enhances compensatory responses to herbivory in wild lima bean. Front Plant Sci.

[CR25] Cameron DD, Neal AL, van Wees SC, Ton J (2013). Mycorrhiza-induced resistance: more than the sum of its parts?. Trends Plant Sci.

[CR26] Chen YH, Gols R, Benrey B (2015). Crop domestication and its impact on naturally selected trophic interactions. Annu Rev Entomol.

[CR27] Chidawanyika F, Muriithi B, Niassy S, Ouya FO, Pittchar JO, Kassie M, Khan ZR (2023). Sustainable intensification of vegetable production using the cereal ‘push-pull technology’: benefits and one health implications. Environm Sust.

[CR28] Chrétien LTS, van der Heide H, Greenberg LO, Giron D, Dicke M, Lucas-Barbosa D (2021). Multiple attack to inflorescences of an annual plant does not interfere with the attraction of parasitoids and pollinators. J Chem Ecol.

[CR29] Conrath U, Beckers GJM, Flors V, García-Agustín P, Jakab G, Mauch F, Newman M-A, Pieterse CMJ, Poinssot B, Pozo MJ, Pugin A, Schaffrath U, Ton J, Wendehenne D, Zimmerli L, Mauch-Mani B (2006). Priming: getting ready for battle. MPMI.

[CR30] Croijmans L, Valstar RT, Schuur L, Jacobs I, van Apeldoorn DF, Poelman EH (2022). Intraspecific plant variation and nonhost herbivores affect parasitoid host location behaviour. Anim Behav.

[CR31] Degenhardt J, Hiltpold I, Köllner TG, Frey M, Gierl A, Gershenzon J, Hibbard BE, Ellersieck MR, Turlings TCJ (2009). Restoring a maize root signal that attracts insect-killing nematodes to control a major pest. Proc Natl Acad Sci USA.

[CR32] de Vries J, Evers JB, Poelman EH (2017). Dynamic plant-plant herbivore interactions govern plant growth-defence integration. Trends Plant Sci.

[CR33] de Vries J, Evers JB, Dicke M, Poelman EH (2019). Ecological interactions shape the adaptive value of plant defence: herbivore attack versus competition for light. Funct Ecol.

[CR34] Divekar PA, Narayana S, Divekar BA, Kumar R, Gadratagi BG, Ray A, Singh AK, Rani V, Singh V, Singh AK, Kumar A, Singh RP, Meena RS, Behera TK (2022). Plant secondary metabolites as defense tools against herbivores for sustainable crop protection. Int J Mol Sci.

[CR35] Dong Y, Jing M, Shen D, Wang C, Zhang M, Liang D, Nyawira KT, Xia Q, Zuo K, Wu S, Wu Y, Dou D, Xia A (2020). The mirid bug Apolygus lucorum deploys a glutathione peroxidase as a candidate effector to enhance plant susceptibility. J Exp Bot.

[CR36] Douma JC, Vermeulen PJ, Poelman EH, Dicke M, Anten NPR (2017). When does it pay to prime for defense? A modeling analysis. New Phytol.

[CR37] Duran-Flores D, Heil M (2016). Sources of specificity in plant damaged-self recognition. Curr Opin Plant Biol.

[CR38] Espinosa-García FJ (2022). The role of phytochemical diversity in the management of agroecosystems. Botan Sci.

[CR39] Erb M, Reymond P (2019). Molecular interactions between plants and insect herbivores. Annu Rev Plant Biol.

[CR40] Erb M (2018). Plant defenses against herbivory: Closing the fitness gap. Trends Plant Sci.

[CR41] Fernandez de Bobadilla M, Vitiello A, Erb M, Poelman EH (2022). Plant defense strategies against attack by multiple herbivores. Trends Plant Sci.

[CR42] Fijen TPM, Scheper JA, Boom TM, Janssen N, Raemakers I, Kleijn D (2018). Insect pollination is at least as important for marketable crop yield as plant quality in a seed crop. Ecol Lett.

[CR43] Frost CJ, Appel HM, Carlson JE, De Moraes CM, Mescher MC, Schultz JC (2007). Within-plant signalling via volatiles overcomes vascular constraints on systemic signalling and primes responses against herbivores. Ecol Lett.

[CR44] Fukami T (2015). Historical Contingency in Community Assembly: Integrating Niches, Species Pools, and Priority Effects. Annu Rev Ecol Evol Syst.

[CR45] Gagic V, Riggi LG, Ekbom B, Malsher G, Rusch A, Bommarco R (2016). Interactive effects of pests increase seed yield. Ecol Evol.

[CR46] Gallai N, Salles JM, Settele J, Vaissière BE (2009). Economic valuation of the vulnerability of world agriculture confronted with pollinator decline. Ecol Econ.

[CR47] Garcia LC, Eubanks MD (2019). Overcompensation for insect herbivory: a review and meta-analysis of the evidence. Ecology.

[CR48] Garcia A, Martinez M, Diaz I, Santamaria ME (2021). The Price of the Induced Defense Against Pests: A Meta-Analysis. Front Plant Sci.

[CR49] Garibaldi LA, Steffan-Dewenter I, Kremen C, Morales JM, Bommarco R, Cunningham SA, Carvalheiro LG, Chacoff NP, Dudenhöffer JH, Greenleaf SS, Holzschuh A, Isaacs R, Krewenka K, Mandelik Y, Mayfield MM, Morandin LA, Potts SG, Ricketts TH, Szentgyörgyi H, Viana BF, Westphal C, Winfree R, Klein AM (2011). Stability of pollination services decreases with isolation from natural areas despite honey bee visits. Ecol Lett.

[CR50] Garibaldi LA, Aizen MA, Klein AM, Cunningham SA, Harder LD (2011). Global growth and stability of agricultural yield decrease with pollinator dependence. Proc Natl Acad Sci USA.

[CR51] Godschalx AL, Stady L, Watzig B, Ballhorn DJ (2016). Is protection against florivory consistent with the optimal defense hypothesis?. BMC Plant Biol.

[CR52] Griese E, Caarls L, Bassetti N, Mohammadin S, Verbaarschot P, Bukovinszkine’Kiss G, Poelman EH, Gols R, Schranz ME, Fatouros NE,  (2021). Insect egg-killing: a new front on the evolutionary arms-race between brassicaceous plants and pierid butterflies. New Phytol.

[CR53] Haddad NM, Crutsinger GM, Gross K, Haarstad J, Tilman D (2011). Plant diversity and the stability of foodwebs. Ecol Lett.

[CR54] Heil M (2002). Ecological costs of induced resistance. Curr Opin Plant Biol.

[CR55] Herms DA, Mattson WJ (1992). The dilemma of plants: To grow or defend. Quarterly Rev Biol.

[CR56] Howe GA, Jander G (2008). Plant immunity to insect herbivores. Annu Rev Plant Biol.

[CR57] Johnson MTJ, Campbell SA, Barrett SCH (2015). Evolutionary Interactions Between Plant Reproduction and Defense Against Herbivores. Annu Rev Ecol Evol Syst.

[CR58] Kaiser L, Ode P, van Nouhuys S, Calatayud P-A, Colazza S, Cortesero A-M, Thiel A, van Baaren J (2017). Chapter Six - The Plant as a Habitat for Entomophagous Insects. Adv Bot Res.

[CR59] Kalske A, Shiojiri K, Uesugi A, Sakata Y, Morrell K, Kessler A (2019). Insect herbivory selects for volatile-mediated plant-plant communication. Curr Biol.

[CR60] Karasov TL, Chae E, Herman JJ, Bergelson J (2017). Mechanisms to mitigate the trade-off between growth and defense. Plant Cell.

[CR61] Karban R (2020). The ecology and evolution of induced responses to herbivory and how plants perceive risk. Ecol Entomol.

[CR62] Karban R, Baldwin IT (1997). Induced Responses to Herbivory.

[CR63] Karban R, Orrock JL (2018). A judgment and decision-making model for plant behavior. Ecology.

[CR64] Karban R, Yang LH (2020). Feeding and damage-induced volatile cues make beetles disperse and produce a more even distribution of damage for sagebrush. J Anim Ecol.

[CR65] Kessler A (2015). The information landscape of plant constitutive and induced secondary metabolite production. Curr Opin Insect Sci.

[CR66] Kessler A, Baldwin IT (2004). Herbivore-induced plant vaccination. Part I. The orchestration of plant defenses in nature and their fitness consequences in the wild tobacco *Nicotiana attenuata*. Plant J.

[CR67] Kessler A, Halitschke R (2007). Specificity and complexity: the impact of herbivore-induced plant responses on arthropod community structure. Curr Opin Plant Biol.

[CR68] Kessler A, Chautá A (2020). The ecological consequences of herbivore-induced plant responses on plant–pollinator interactions. Emerg Top Life Sci.

[CR69] Kessler D, Baldwin IT (2007). Making sense of nectar scents: the effects of nectar secondary metabolites on floral visitors of Nicotiana attenuata. Plant J.

[CR70] Kessler A, Kalske A (2018). Plant secondary metabolite diversity and species interactions. Annu Rev Ecol Evol Syst.

[CR71] Khan Z, Midega C, Pittchar J, Pickett J, Bruce T (2011). Push—pull technology: a conservation agriculture approach for integrated management of insect pests, weeds and soil health in Africa. Internl J Agricult Sustain.

[CR72] Khan Z, Midega CA, Hooper A, Pickett J (2016). Push-pull: chemical ecology-based integrated pest management technology. J Chem Ecol.

[CR73] Koski TM, de Jong S, Muola A, Amby DB, Andreasson E, Stenberg JA (2021). ‘Resistance mixtures’ reduce insect herbivory in Strawberry (*Fragaria vesca*) plantations. Front Plant Sci.

[CR74] Kovács-Hostyánszki A, Espíndola A, Vanbergen AJ, Settele J, Kremen C, Dicks LV (2017). Ecological intensification to mitigate impacts of conventional intensive land use on pollinators and pollination. Ecol Lett.

[CR75] Kunert G, Reinhold C, Gershenzon J (2010). Constitutive emission of the aphid alarm pheromone, (E)-β-farnesene, from plants does not serve as a direct defense against aphids. BMC Ecol.

[CR76] Lee Díaz AS, Macheda D, Saha H, Ploll U, Orine D, Biere A (2021). Tackling the Context-Dependency of Microbial-Induced Resistance. Agronomy.

[CR77] Li X, Garvey M, Kaplan I, Li B, Carrillo J (2018). Domestication of tomato has reduced the attraction of herbivore natural enemies to pest-damaged plants. Agr Forest Entomol.

[CR78] Malook SU, Maqbool S, Hafeez M, Karunarathna SC, Suwannarach N (2022). Molecular and biochemical mechanisms of elicitors in pest resistance. Life.

[CR79] Malone S, Menalled F, Weaver D, Seipel T, Hofland M, Runyon J, Bourgault M, Boss DL, Trowbridge A (2022). Cropping systems alter plant volatile emissions in the field through soil legacy effects. Renew Agricult Food Syst.

[CR80] Mathur V, Wagenaar R, Caissard J-C, Reddy AS, Vet LEM, Cortesero A-M, van Dam NM (2013). A novel indirect defence in Brassicaceae: Structure and function of extrafloral nectaries in *Brassica juncea*. Plant Cell Environ.

[CR81] Mauch-Mani B, Baccelli I, Luna E, Flors V (2017). Defense Priming: An Adaptive Part of Induced Resistance. Annu Rev Plant Biol.

[CR82] McArt SH, Halitschke R, Salminen J, Thaler JS (2013). Leaf herbivory increases plant fitness via induced resistance to seed predators. Ecology.

[CR83] Mertens D, Boege K, Kessler A, Koricheva J, Thaler JS, Whiteman NK, Poelman EH (2021). Predictability of biotic stress structures plant defence evolution. Trends Ecol Evol.

[CR84] Mertens D, Fernández de Bobadilla M, Rusman Q, Bloem J, Douma JC, Poelman EH (2021). Plant defence to sequential attack is adapted to prevalent herbivores. Nat Plants.

[CR85] Mertens D, Douma JC, Kamps BBJ, Zhu Y, Zwartsenberg SA, Poelman EH (2022) Priority effects in herbivore communities vary in effect on plant development and reproduction in four Brassicaceae plant species. BioRxiv 2022.12.10.519923 10.1101/2022.12.10.519923

[CR86] Minard G, Kahilainen A, Biere A, Pakkanen H, Mappes J, Saastamoinen M (2022). Complex plant quality—microbiota–population interactions modulate the response of a specialist herbivore to the defence of its host plant. Funct Ecol.

[CR87] Moreira X, Abdala-Roberts L (2019). Specificity and context-dependency of plant–plant communication in response to insect herbivory. Curr Opin Insect Sci.

[CR88] Moreira X, Abdala-Roberts L, Gols R, Francisco M (2018). Plant domestication decreases both constitutive and induced chemical defences by direct selection against defensive traits. Sci Rep.

[CR89] Morrell K, Kessler A (2017). Plant communication in a widespread goldenrod: keeping herbivores on the move. Funct Ecol.

[CR90] Muola A, Weber D, Malm LE, Egan PA, Glinwood R, Parachnowitsch AL, Stenberg JA (2017). Direct and pollinator-mediated effects of herbivory on strawberry and the potential for improved resistance. Front Plant Sci.

[CR91] Nicholls CI, Altieri MA (2013). Plant biodiversity enhances bees and other insect pollinators in agroecosystems. A Review Agron Sustain Develop.

[CR92] Nguyen D, Rieu I, Mariani C, van Dam NM (2016). How plants handle multiple stresses: hormonal interactions underlying responses to abiotic stress and insect herbivory. Plant Mol Biol.

[CR93] Omer AD, Thaler JS, Granett J, Karban R (2000). Jasmonic Acid induced resistance in Grapevines to a root and leaf feeder. J Econom Entomol.

[CR94] Pappas ML, Steppuhn A, Geuss D, Topalidou N, Zografou A, Sabelis MW, Broufas GD (2015). Beyond predation: The zoophytophagous predator Macrolophus pygmaeus induces Tomato resistance against Spider Mites. PLoS ONE.

[CR95] Pappas ML, Broekgaarden C, Broufas GD, Kant MR, Messelink GJ, Steppuhn A, Wäckers F, van Dam NM (2017). Induced plant defences in biological control of arthropod pests: a double-edged sword. Pest Manag Sci.

[CR96] Pearse IS, LoPresti E, Schaeffer RN, Wetzel WC, Mooney KA, Ali JG, Ode PJ, Eubanks MD, Bronstein JL, Weber MG (2020). Generalising indirect defence and resistance of plants. Ecol Lett.

[CR97] Petrén H, Anaia RA, Aragam KS, Bräutigam A, Eckert S, Heinen R, Jakobs R, Ojeda-Prieto L, Popp M, Sasidharan R, Schnitzler J-P, Steppuhn A, Thon F, Tschikin S, Unsicker SB, van Dam NM, Weisser WW, Wittmann MJ, Yepes S, Ziaja D, Müller C, Junker RR (2023) Understanding the phytochemical diversity of plants: Quantification, variation and ecological function. bioRxiv 2023.03.23.533415. 10.1101/2023.03.23.533415

[CR98] Pieterse CMJ, Van der Does D, Zamioudis C, Leon-Reyes A, Van Wees SCM (2012). Hormonal Modulation of Plant Immunity. Annu Rev Cell Developm Biol.

[CR99] Poelman EH, Broekgaarden C, van Loon JJA, Dicke M (2008). Early season herbivore differentially affects plant defence responses to subsequently colonizing herbivores and their abundance in the field. Mol Ecol.

[CR100] Poelman EH, Oduor AMO, Broekgaarden C, Hordijk CA, Jansen JJ, Van Loon JJA, Van Dam NM, Vet LEM, Dicke M (2009). Field parasitism rates of caterpillars on Brassica oleracea plants are reliably predicted by differential attraction of Cotesia parasitoids. Funct Ecol.

[CR101] Poelman EH (2015). From induced resistance to defence in plant-insect interactions. Entomol Exp Appl.

[CR102] Poelman EH, Kessler A (2016). Keystone herbivores and the evolution of plant defenses. Trends Plant Sci.

[CR103] Poveda K, Jiménez MIG, Kessler A (2010). The enemy as ally: herbivore-induced increase in crop yield. Ecol Appl.

[CR104] Rubin IN, Ellner SP, Kessler A, Morrell KA (2015). Informed herbivore movement and interplant communication determine the effects of induced resistance in an individual-based model. J Anim Ecol.

[CR105] Rusman Q, Poelman EH, Nowrin F, Polder G, Lucas-Barbosa D (2019). Floral plasticity: Herbivore-species-specific-induced changes in flower traits with contrasting effects on pollinator visitation. Plant Cell Environ.

[CR106] Sapir G, Baras Z, Azmon G, Goldway M, Shafir S, Allouche A, Stern E, Stern RA (2017). Synergistic effects between bumblebees and honey bees in apple orchards increase cross pollination, seed number and fruit size. Scientia Horticult.

[CR107] Schuman MC, Baldwin IT (2016). The layers of plant responses to insect herbivores. Annu Rev Entomol.

[CR108] Schuman MC, Barthel K, Baldwin IT (2012). Herbivory-induced volatiles function as defenses increasing fitness of the native plant Nicotiana attenuata in nature. ELife.

[CR109] Selig P, Keough S, Nalam VJ, Nachappa P (2016). Jasmonate-dependent plant defenses mediate soybean thrips and soybean aphid performance on soybean. Arthropod-Plant Int.

[CR110] Singaravelan N, Nee’man G, Inbar M, Izhaki I (2005). Feeding responses of free-flying honeybees to secondary compounds mimicking floral nectars. J Chem Ecol.

[CR111] Stam JM, Kroes A, Li Y, Gols R, van Loon JJA, Poelman EH, Dicke M (2014). Plant Interactions with Multiple Insect Herbivores: From Community to Genes. Annu Rev Plant Biol.

[CR112] Stanley DA, Garratt MPD, Wickens JB, Wickens VJ, Potts SG, Raine NE (2015). Neonicotinoid pesticide exposure impairs crop pollination services provided by bumblebees. Nature.

[CR113] Stevenson PC, Nicolson SW, Wright GA (2017). Plant secondary metabolites in nectar: impacts on pollinators and ecological functions. Funct Ecol.

[CR114] Stenberg JA, Heil M, Åhman I, Björkman C (2015). Optimizing Crops for Biocontrol of Pests and Disease. Trends Plant Sci.

[CR115] Stork W, Diezel C, Halitschke R, van Gális I, Baldwin IT (2009). An Ecological Analysis of the Herbivory-Elicited JA Burst and Its Metabolism: Plant Memory Processes and Predictions of the Moving Target Model. PLoS ONE.

[CR116] Strauss SY, Rudgers JA, Lau JA, Irwin RA (2002). Direct and ecological costs of resistance to herbivory. Trends Ecol Evol.

[CR117] Tan C-W, Peiffer M, Hoover K, Rosa C, Acevedo FE, Felton GW (2018). Symbiotic polydnavirus of a parasite manipulates caterpillar and plant immunity. Proc Natl Acad Sci.

[CR118] Tan C-W, Peiffer M, Hoover K, Rosa C, Felton GW (2019). Parasitic wasp mediates plant perception of insect herbivores. J Chem Ecol.

[CR119] Thomson JD, Draguleasa MA, Tan MG (2015). Flowers with caffeinated nectar receive more pollination. Arthropod-Plant Inter.

[CR120] Veyrat N, Robert CAM, Turlings TCJ, Erb M (2016). Herbivore intoxication as a potential primary function of an inducible volatile plant signal. J Ecol.

[CR121] Vos IA, Pieterse CMJ, van Wees SCM (2013). Costs and benefits of hormone-regulated plant defences. Plant Pathol.

[CR122] War AR, Paulraj MG, War MY, Ignacimuthu S (2011). Jasmonic acid-mediated-induced resistance in groundnut (Arachis hypogaea L.) against *Helicoverpa armigera* (Hubner)(Lepidoptera: Noctuidae). J Plant Growth Reg.

[CR123] Whitehead SR, Turcotte MM, Poveda K (2017). Domestication impacts on plant–herbivore interactions: a meta-analysis. Philosophical Transact Royal Soc B: Biol Sci.

[CR124] Wright GA, Baker DD, Palmer MJ, Stabler D, Mustard JA, Power EF, Borland AM, Stevenson PC (2013). Caffeine in floral nectar enhances a pollinator’s memory of reward. Science.

[CR125] Zangerl A (2003). Evolution of induced plant responses to herbivores. Basic Appl Ecol.

[CR126] Zangerl AR, Rutledge CE (1996). The probability of attack and patterns of constitutive and induced defense: A test of optimal defense theory. Am Nat.

[CR127] Zhang Y, Liu Y, Liang X, Wu C, Liu X, Wu M, Yao X, Qiao Y, Zhan X, Chen Q (2023). Exogenous methyl jasmonate induced cassava defense response and enhanced resistance to *Tetranychus urticae*. Exp Appl Acarol.

[CR128] Zhu F, Poelman EH, Dicke M (2018). Insect herbivore-associated organisms affect plant responses to herbivory. New Phytol.

[CR129] Zhu F, Cusumano A, Bloem J, Weldegergis BT, Villela A, Fatouros NE, van Loon JJA, Dicke M, Harvey JA, Vogel H, Poelman EH (2018). Symbiotic polydnavirus and venom reveal parasitoid to its hyperparasitoids. Proc Natl Acad Sci.

